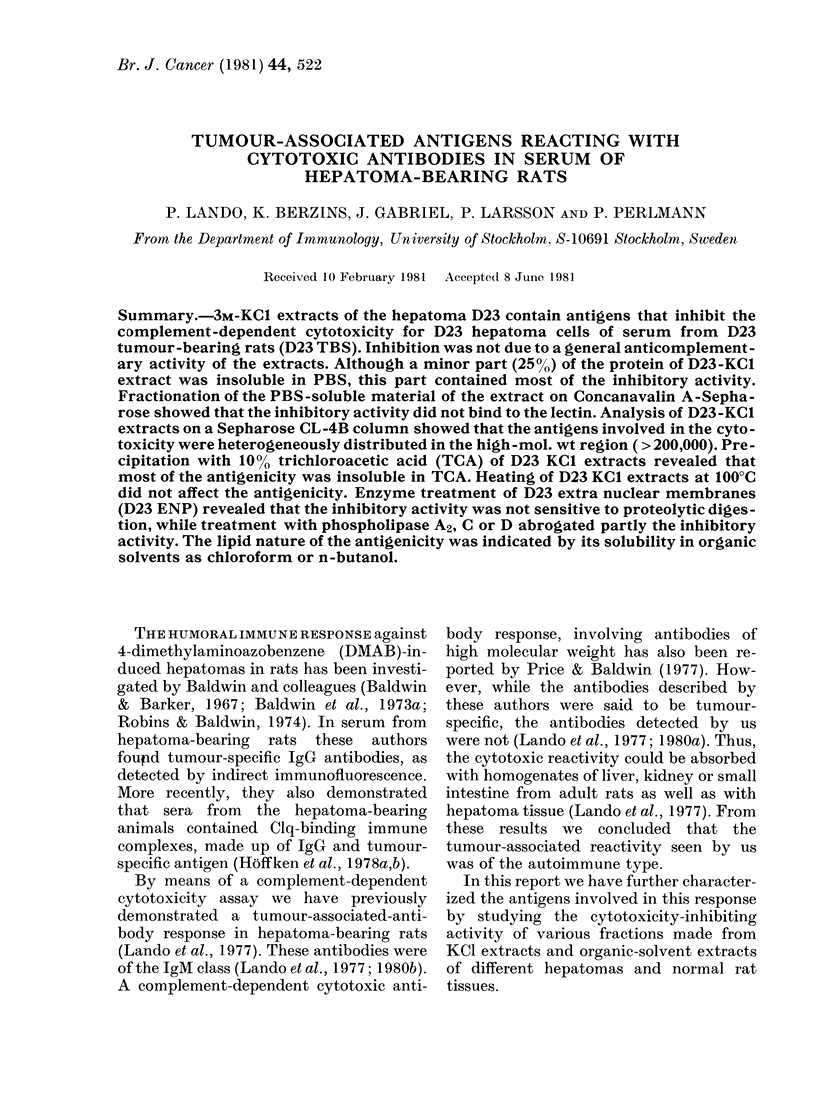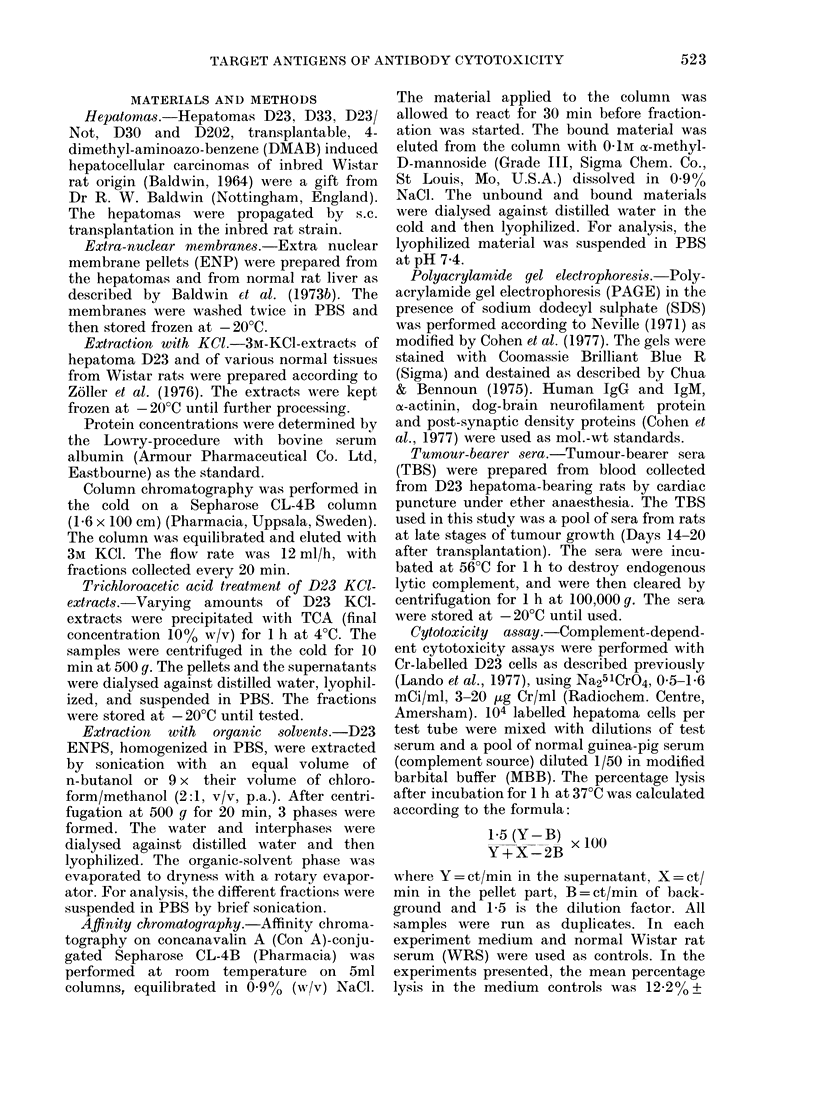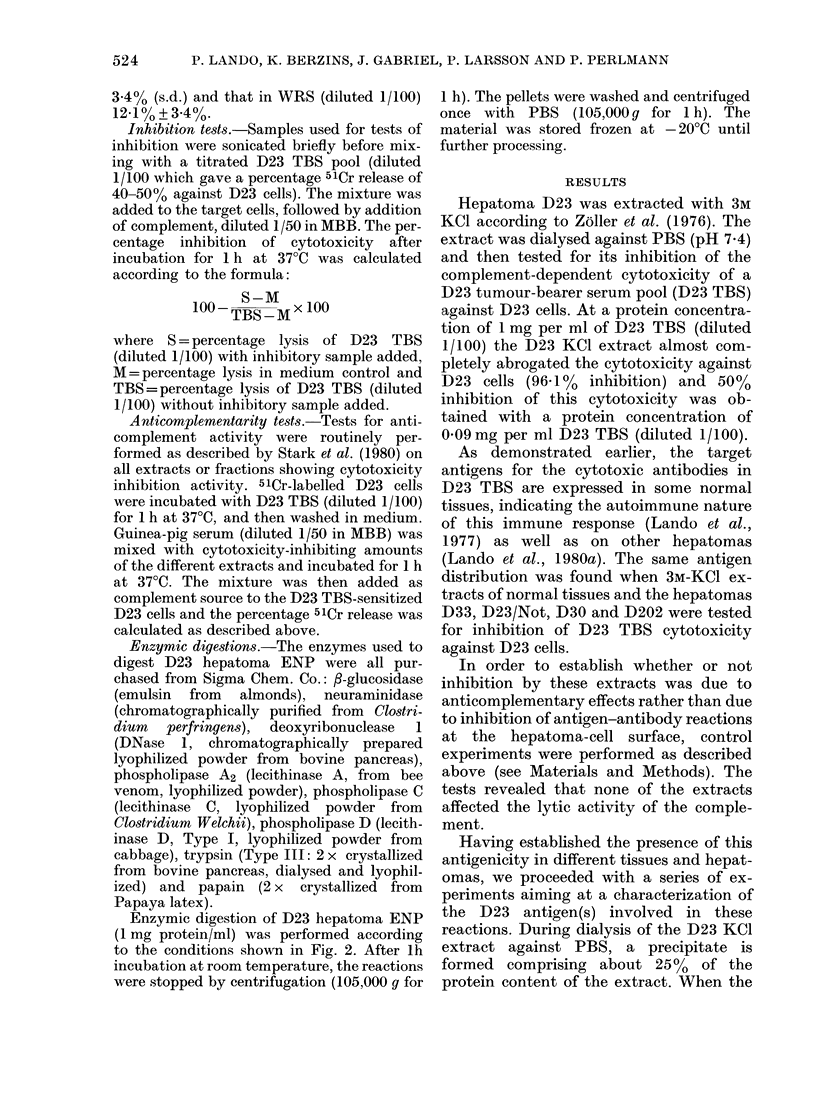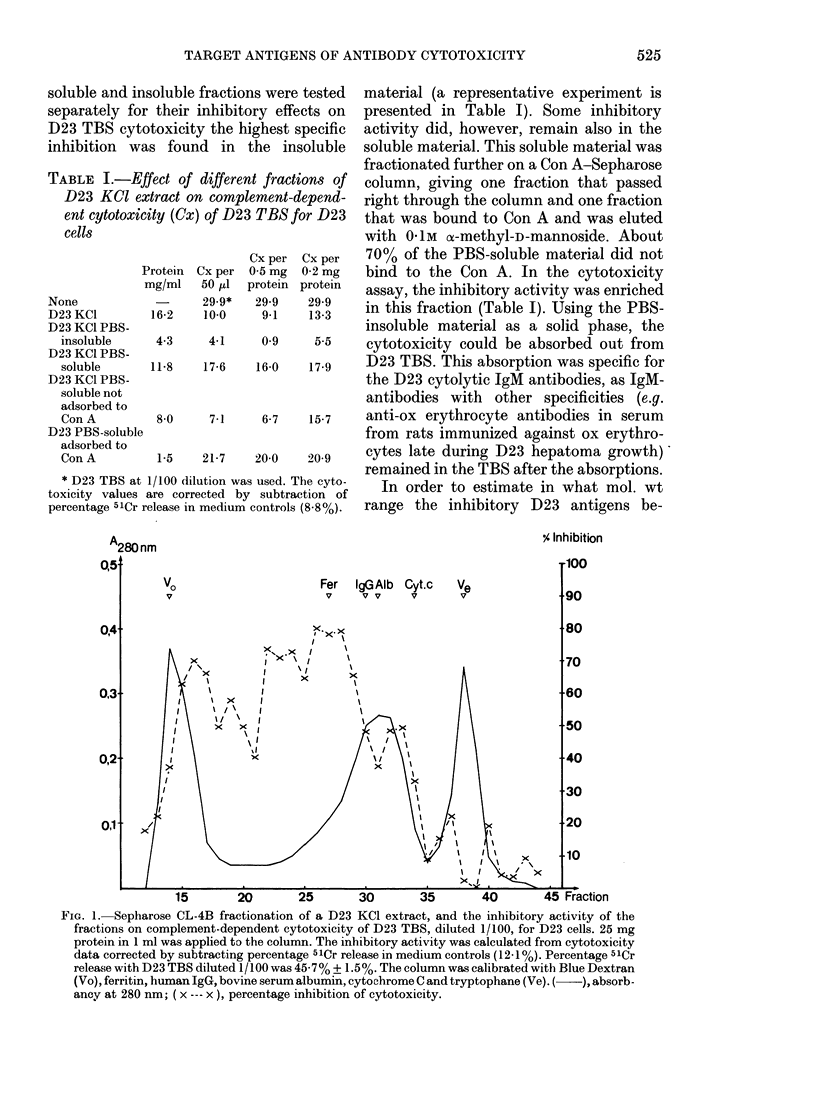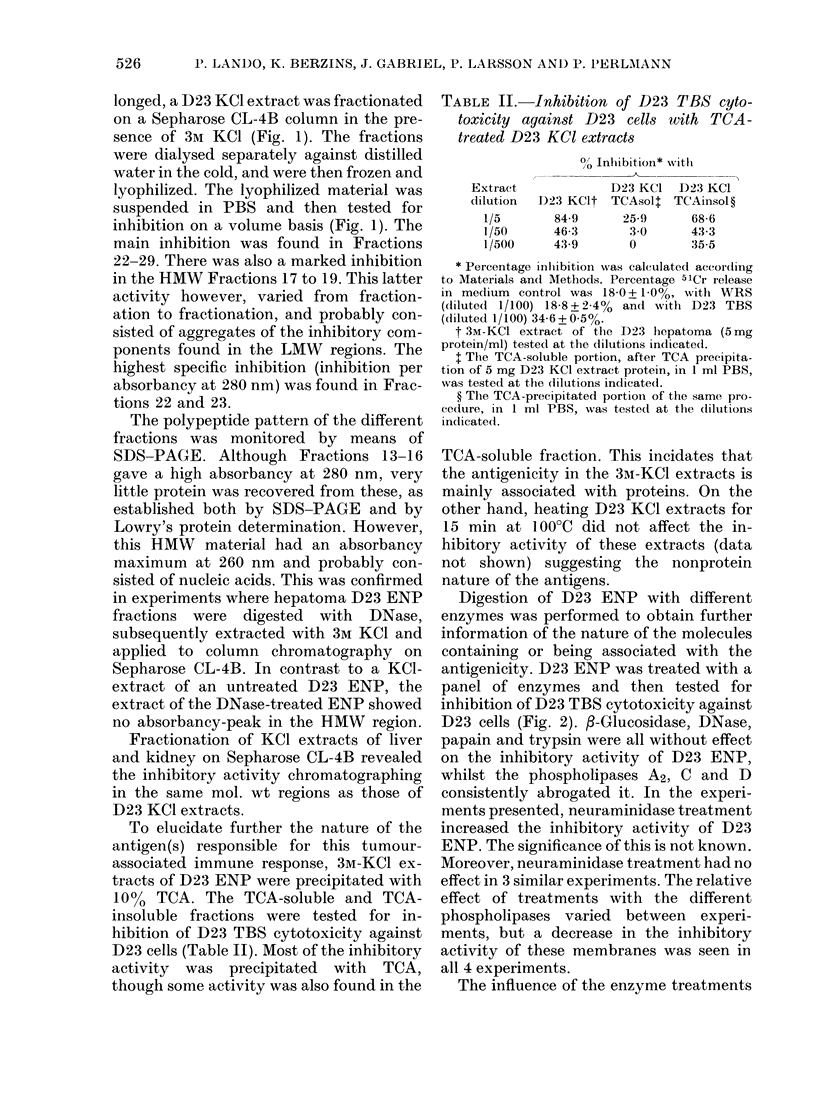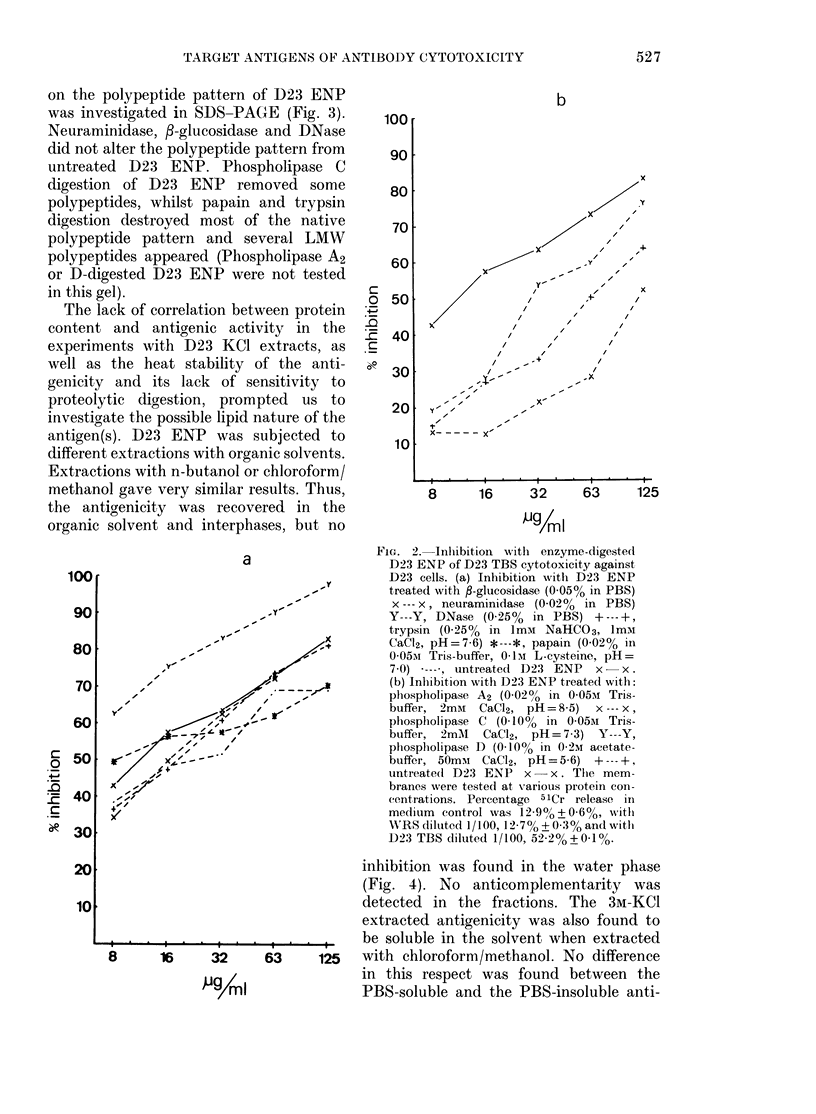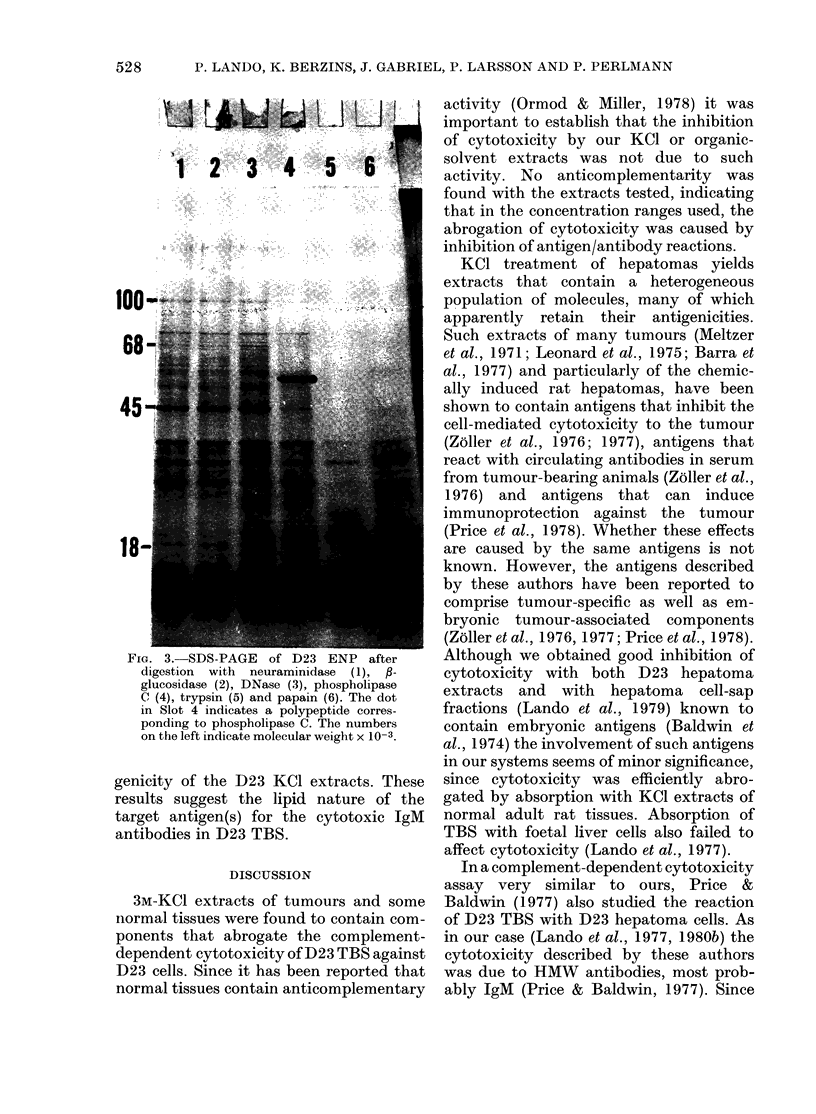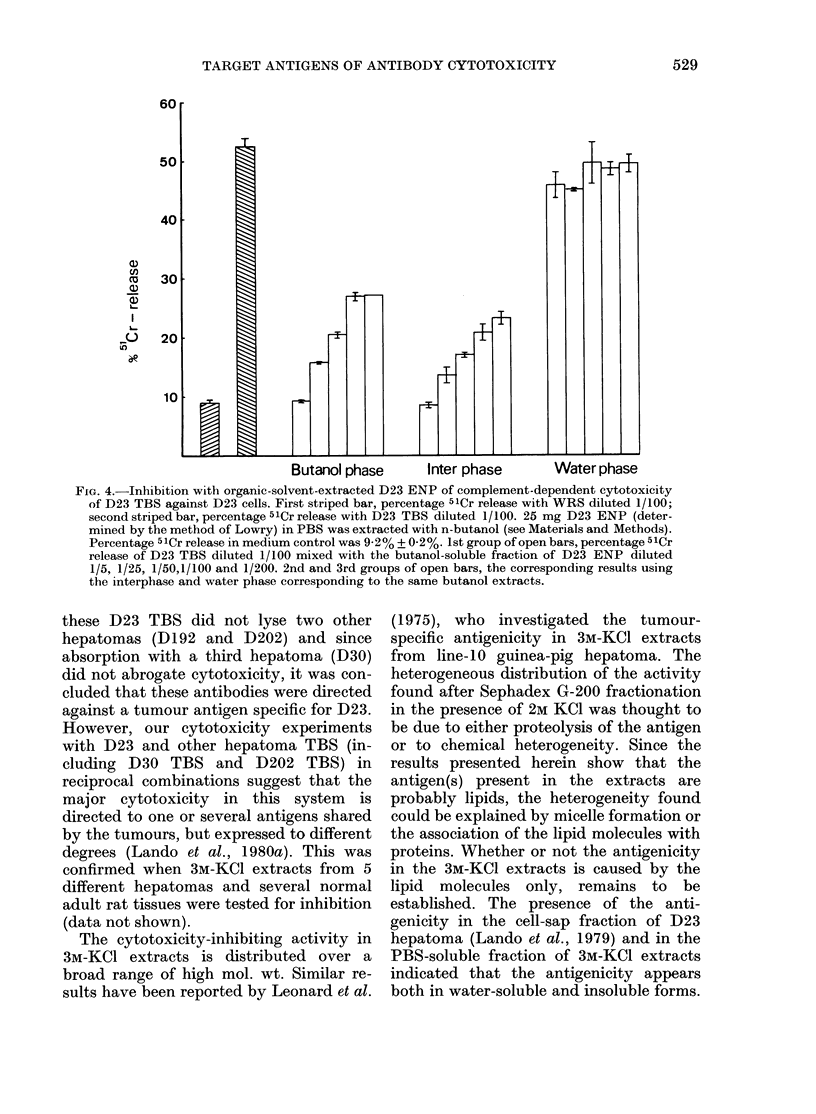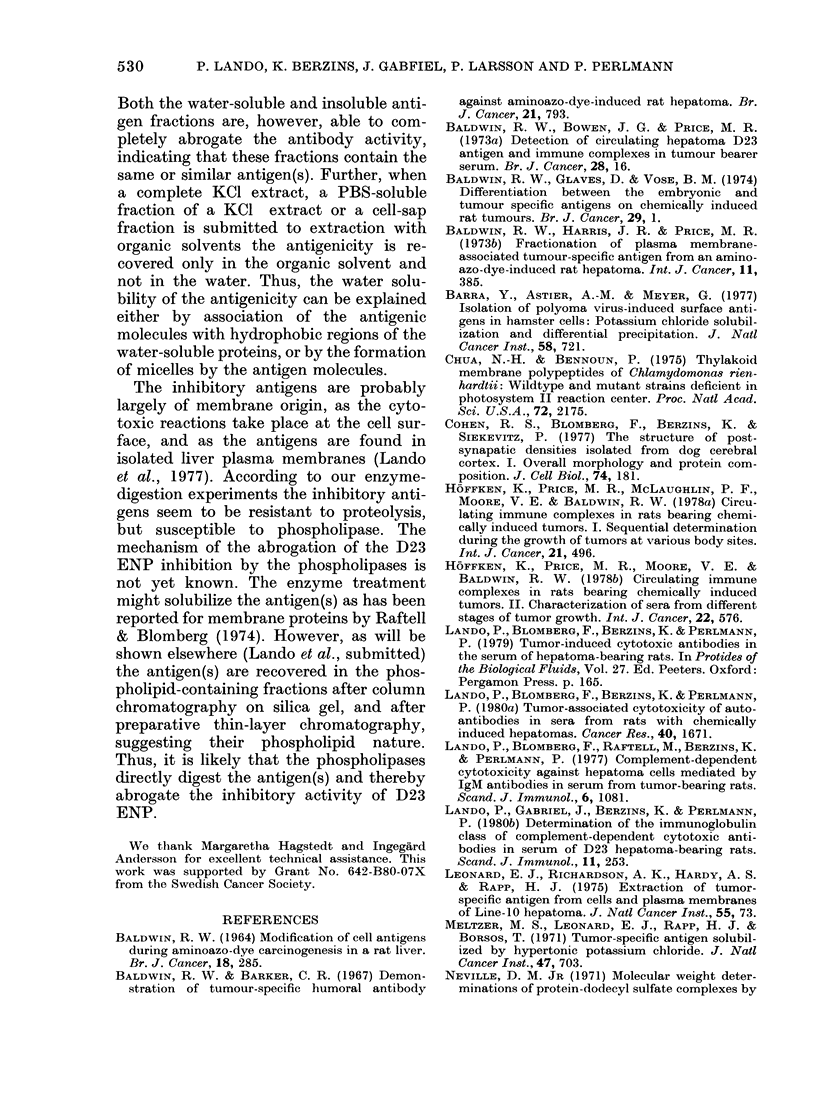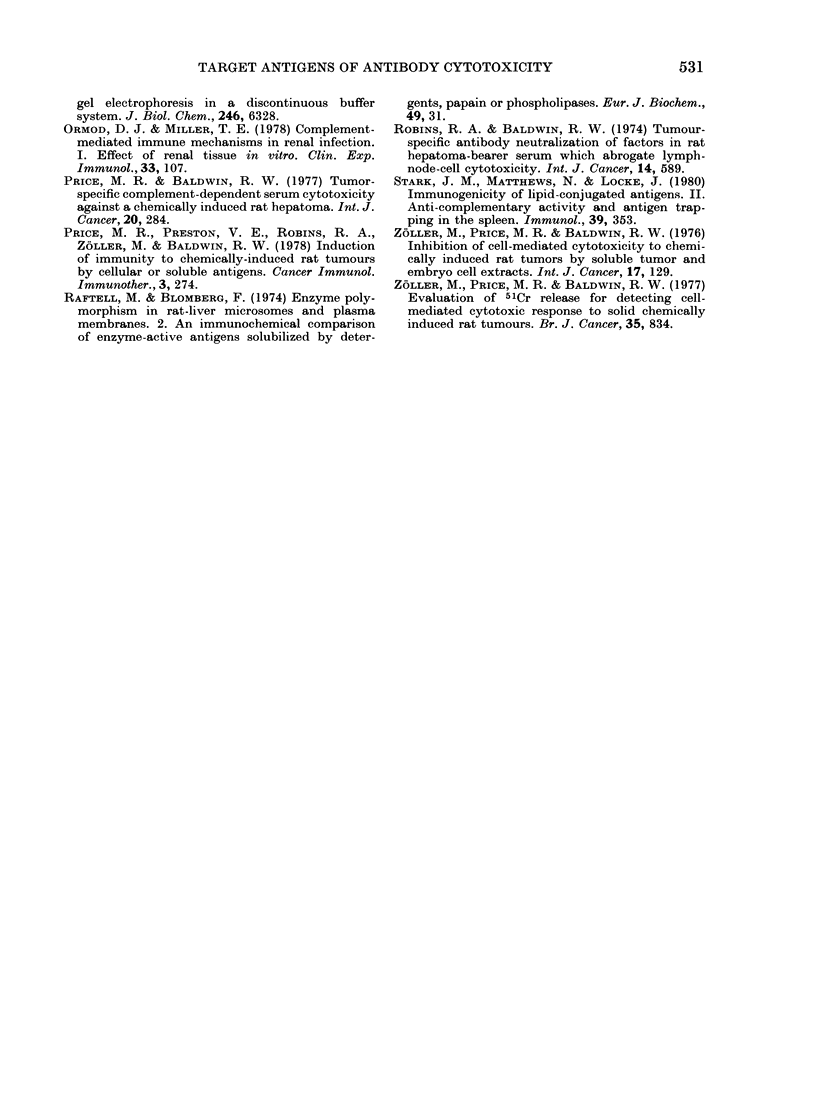# Tumour-associated antigens reacting with cytotoxic antibodies in serum of hepatoma-bearing rats.

**DOI:** 10.1038/bjc.1981.221

**Published:** 1981-10

**Authors:** P. Lando, K. Berzins, J. Gabriel, P. Larsson, P. Perlmann

## Abstract

**Images:**


*Br. J. Cancer* (1981) **44**, 522

### TUMOUR-ASSOCIATED ANTIGENS REACTING WITH CYTOTOXIC ANTIBODIES IN SERUM OF HEPATOMA-BEARING RATS

P. LANDO, K. BERZINS, J. GABRIEL, P. LARSSON AND P. PERLMANN

*From the Department of Immunology, University of Stockholm, S-10691 Stockholm, Sweden*

Received 10 February 1981 Accepted 8 June 1981

**Summary.—3M-KCl extracts of the hepatoma D23 contain antigens that inhibit the complement-dependent cytotoxicity for D23 hepatoma cells of serum from D23 tumour-bearing rats (D23 TBS). Inhibition was not due to a general anticomplementary activity of the extracts. Although a minor part (25%) of the protein of D23-KCl extract was insoluble in PBS, this part contained most of the inhibitory activity. Fractionation of the PBS-soluble material of the extract on Concanavalin A-Sepharose showed that the inhibitory activity did not bind to the lectin. Analysis of D23-KCl extracts on a Sepharose CL-4B column showed that the antigens involved in the cytotoxicity were heterogeneously distributed in the high-mol. wt region (>200,000). Precipitation with 10% trichloroacetic acid (TCA) of D23 KCl extracts revealed that most of the antigenicity was insoluble in TCA. Heating of D23 KCl extracts at 100°C did not affect the antigenicity. Enzyme treatment of D23 extra nuclear membranes (D23 ENP) revealed that the inhibitory activity was not sensitive to proteolytic digestion, while treatment with phospholipase $A_2$, C or D abrogated partly the inhibitory activity. The lipid nature of the antigenicity was indicated by its solubility in organic solvents as chloroform or n-butanol.**

THE HUMORAL IMMUNE RESPONSE against 4-dimethylaminoazobenzene (DMAB)-induced hepatomas in rats has been investigated by Baldwin and colleagues (Baldwin & Barker, 1967; Baldwin *et al.*, 1973*a*; Robins & Baldwin, 1974). In serum from hepatoma-bearing rats these authors found tumour-specific IgG antibodies, as detected by indirect immunofluorescence. More recently, they also demonstrated that sera from the hepatoma-bearing animals contained Clq-binding immune complexes, made up of IgG and tumour-specific antigen (Höffken *et al.*, 1978*a*,*b*).

By means of a complement-dependent cytotoxicity assay we have previously demonstrated a tumour-associated-antibody response in hepatoma-bearing rats (Lando *et al.*, 1977). These antibodies were of the IgM class (Lando *et al.*, 1977; 1980*b*). A complement-dependent cytotoxic antibody response, involving antibodies of high molecular weight has also been reported by Price & Baldwin (1977). However, while the antibodies described by these authors were said to be tumour-specific, the antibodies detected by us were not (Lando *et al.*, 1977; 1980*a*). Thus, the cytotoxic reactivity could be absorbed with homogenates of liver, kidney or small intestine from adult rats as well as with hepatoma tissue (Lando *et al.*, 1977). From these results we concluded that the tumour-associated reactivity seen by us was of the autoimmune type.

In this report we have further characterized the antigens involved in this response by studying the cytotoxicity-inhibiting activity of various fractions made from KCl extracts and organic-solvent extracts of different hepatomas and normal rat tissues.

### MATERIALS AND METHODS

*Hepatomas.*—Hepatomas D23, D33, D23/Not, D30 and D202, transplantable, 4-dimethyl-aminoazo-benzene (DMAB) induced hepatocellular carcinomas of inbred Wistar rat origin (Baldwin, 1964) were a gift from Dr R. W. Baldwin (Nottingham, England). The hepatomas were propagated by s.c. transplantation in the inbred rat strain.

*Extra-nuclear membranes.*—Extra nuclear membrane pellets (ENP) were prepared from the hepatomas and from normal rat liver as described by Baldwin *et al.* (1973*b*). The membranes were washed twice in PBS and then stored frozen at −20°C.

*Extraction with KCl.*—3M-KCl-extracts of hepatoma D23 and of various normal tissues from Wistar rats were prepared according to Zöller *et al.* (1976). The extracts were kept frozen at −20°C until further processing.

Protein concentrations were determined by the Lowry-procedure with bovine serum albumin (Armour Pharmaceutical Co. Ltd, Eastbourne) as the standard.

Column chromatography was performed in the cold on a Sepharose CL-4B column (1·6 × 100 cm) (Pharmacia, Uppsala, Sweden). The column was equilibrated and eluted with 3M KCl. The flow rate was 12 ml/h, with fractions collected every 20 min.

*Trichloroacetic acid treatment of D23 KCl-extracts.*—Varying amounts of D23 KCl-extracts were precipitated with TCA (final concentration 10% w/v) for 1 h at 4°C. The samples were centrifuged in the cold for 10 min at 500 *g*. The pellets and the supernatants were dialysed against distilled water, lyophilized, and suspended in PBS. The fractions were stored at −20°C until tested.

*Extraction with organic solvents.*—D23 ENPS, homogenized in PBS, were extracted by sonication with an equal volume of n-butanol or 9 × their volume of chloroform/methanol (2:1, v/v, p.a.). After centrifugation at 500 *g* for 20 min, 3 phases were formed. The water and interphases were dialysed against distilled water and then lyophilized. The organic-solvent phase was evaporated to dryness with a rotary evaporator. For analysis, the different fractions were suspended in PBS by brief sonication.

*Affinity chromatography.*—Affinity chromatography on concanavalin A (Con A)-conjugated Sepharose CL-4B (Pharmacia) was performed at room temperature on 5ml columns, equilibrated in 0·9% (w/v) NaCl. The material applied to the column was allowed to react for 30 min before fractionation was started. The bound material was eluted from the column with 0·1M α-methyl-D-mannoside (Grade III, Sigma Chem. Co., St Louis, Mo, U.S.A.) dissolved in 0·9% NaCl. The unbound and bound materials were dialysed against distilled water in the cold and then lyophilized. For analysis, the lyophilized material was suspended in PBS at pH 7·4.

*Polyacrylamide gel electrophoresis.*—Polyacrylamide gel electrophoresis (PAGE) in the presence of sodium dodecyl sulphate (SDS) was performed according to Neville (1971) as modified by Cohen *et al.* (1977). The gels were stained with Coomassie Brilliant Blue R (Sigma) and destained as described by Chua & Bennoun (1975). Human IgG and IgM, α-actinin, dog-brain neurofilament protein and post-synaptic density proteins (Cohen *et al.*, 1977) were used as mol.-wt standards.

*Tumour-bearer sera.*—Tumour-bearer sera (TBS) were prepared from blood collected from D23 hepatoma-bearing rats by cardiac puncture under ether anaesthesia. The TBS used in this study was a pool of sera from rats at late stages of tumour growth (Days 14–20 after transplantation). The sera were incubated at 56°C for 1 h to destroy endogenous lytic complement, and were then cleared by centrifugation for 1 h at 100,000 *g*. The sera were stored at −20°C until used.

*Cytotoxicity assay.*—Complement-dependent cytotoxicity assays were performed with Cr-labelled D23 cells as described previously (Lando *et al.*, 1977), using $Na_2{}^{51}CrO_4$, 0·5–1·6 mCi/ml, 3–20 μg Cr/ml (Radiochem. Centre, Amersham). $10^4$ labelled hepatoma cells per test tube were mixed with dilutions of test serum and a pool of normal guinea-pig serum (complement source) diluted 1/50 in modified barbital buffer (MBB). The percentage lysis after incubation for 1 h at 37°C was calculated according to the formula:

$$\frac{1{\cdot}5\,(Y-B)}{Y+X-2B}\times 100$$

where Y = ct/min in the supernatant, X = ct/min in the pellet part, B = ct/min of background and 1·5 is the dilution factor. All samples were run as duplicates. In each experiment medium and normal Wistar rat serum (WRS) were used as controls. In the experiments presented, the mean percentage lysis in the medium controls was 12·2% ±

3·4% (s.d.) and that in WRS (diluted 1/100) 12·1% ± 3·4%.

*Inhibition tests.*—Samples used for tests of inhibition were sonicated briefly before mixing with a titrated D23 TBS pool (diluted 1/100 which gave a percentage $^{51}$Cr release of 40–50% against D23 cells). The mixture was added to the target cells, followed by addition of complement, diluted 1/50 in MBB. The percentage inhibition of cytotoxicity after incubation for 1 h at 37°C was calculated according to the formula:

$$100 - \frac{S - M}{TBS - M} \times 100$$

where S = percentage lysis of D23 TBS (diluted 1/100) with inhibitory sample added, M = percentage lysis in medium control and TBS = percentage lysis of D23 TBS (diluted 1/100) without inhibitory sample added.

*Anticomplementarity tests.*—Tests for anti-complement activity were routinely performed as described by Stark *et al.* (1980) on all extracts or fractions showing cytotoxicity inhibition activity. $^{51}$Cr-labelled D23 cells were incubated with D23 TBS (diluted 1/100) for 1 h at 37°C, and then washed in medium. Guinea-pig serum (diluted 1/50 in MBB) was mixed with cytotoxicity-inhibiting amounts of the different extracts and incubated for 1 h at 37°C. The mixture was then added as complement source to the D23 TBS-sensitized D23 cells and the percentage $^{51}$Cr release was calculated as described above.

*Enzymic digestions.*—The enzymes used to digest D23 hepatoma ENP were all purchased from Sigma Chem. Co.: β-glucosidase (emulsin from almonds), neuraminidase (chromatographically purified from *Clostridium perfringens*), deoxyribonuclease 1 (DNase 1, chromatographically prepared lyophilized powder from bovine pancreas), phospholipase $A_2$ (lecithinase A, from bee venom, lyophilized powder), phospholipase C (lecithinase C, lyophilized powder from *Clostridium Welchii*), phospholipase D (lecithinase D, Type I, lyophilized powder from cabbage), trypsin (Type III: 2× crystallized from bovine pancreas, dialysed and lyophilized) and papain (2× crystallized from Papaya latex).

Enzymic digestion of D23 hepatoma ENP (1 mg protein/ml) was performed according to the conditions shown in Fig. 2. After 1h incubation at room temperature, the reactions were stopped by centrifugation (105,000 *g* for 1 h). The pellets were washed and centrifuged once with PBS (105,000 *g* for 1 h). The material was stored frozen at −20°C until further processing.

### RESULTS

Hepatoma D23 was extracted with 3M KCl according to Zöller *et al.* (1976). The extract was dialysed against PBS (pH 7·4) and then tested for its inhibition of the complement-dependent cytotoxicity of a D23 tumour-bearer serum pool (D23 TBS) against D23 cells. At a protein concentration of 1 mg per ml of D23 TBS (diluted 1/100) the D23 KCl extract almost completely abrogated the cytotoxicity against D23 cells (96·1% inhibition) and 50% inhibition of this cytotoxicity was obtained with a protein concentration of 0·09 mg per ml D23 TBS (diluted 1/100).

As demonstrated earlier, the target antigens for the cytotoxic antibodies in D23 TBS are expressed in some normal tissues, indicating the autoimmune nature of this immune response (Lando *et al.*, 1977) as well as on other hepatomas (Lando *et al.*, 1980*a*). The same antigen distribution was found when 3M-KCl extracts of normal tissues and the hepatomas D33, D23/Not, D30 and D202 were tested for inhibition of D23 TBS cytotoxicity against D23 cells.

In order to establish whether or not inhibition by these extracts was due to anticomplementary effects rather than due to inhibition of antigen–antibody reactions at the hepatoma-cell surface, control experiments were performed as described above (see Materials and Methods). The tests revealed that none of the extracts affected the lytic activity of the complement.

Having established the presence of this antigenicity in different tissues and hepatomas, we proceeded with a series of experiments aiming at a characterization of the D23 antigen(s) involved in these reactions. During dialysis of the D23 KCl extract against PBS, a precipitate is formed comprising about 25% of the protein content of the extract. When the soluble and insoluble fractions were tested separately for their inhibitory effects on D23 TBS cytotoxicity the highest specific inhibition was found in the insoluble material (a representative experiment is presented in Table I). Some inhibitory activity did, however, remain also in the soluble material. This soluble material was fractionated further on a Con A–Sepharose column, giving one fraction that passed right through the column and one fraction that was bound to Con A and was eluted with 0·1M α-methyl-D-mannoside. About 70% of the PBS-soluble material did not bind to the Con A. In the cytotoxicity assay, the inhibitory activity was enriched in this fraction (Table I). Using the PBS-insoluble material as a solid phase, the cytotoxicity could be absorbed out from D23 TBS. This absorption was specific for the D23 cytolytic IgM antibodies, as IgM-antibodies with other specificities (*e.g.* anti-ox erythrocyte antibodies in serum from rats immunized against ox erythrocytes late during D23 hepatoma growth) remained in the TBS after the absorptions.

TABLE I.—*Effect of different fractions of D23 KCl extract on complement-dependent cytotoxicity* (*Cx*) *of D23 TBS for D23 cells*

| | Protein mg/ml | Cx per 50 μl | Cx per 0·5 mg protein | Cx per 0·2 mg protein |
|---|---|---|---|---|
| None | — | 29·9* | 29·9 | 29·9 |
| D23 KCl | 16·2 | 10·0 | 9·1 | 13·3 |
| D23 KCl PBS-insoluble | 4·3 | 4·1 | 0·9 | 5·5 |
| D23 KCl PBS-soluble | 11·8 | 17·6 | 16·0 | 17·9 |
| D23 KCl PBS-soluble not adsorbed to Con A | 8·0 | 7·1 | 6·7 | 15·7 |
| D23 PBS-soluble adsorbed to Con A | 1·5 | 21·7 | 20·0 | 20·9 |

* D23 TBS at 1/100 dilution was used. The cytotoxicity values are corrected by subtraction of percentage $^{51}$Cr release in medium controls (8·8%).

In order to estimate in what mol. wt range the inhibitory D23 antigens be-

FIG. 1.—Sepharose CL-4B fractionation of a D23 KCl extract, and the inhibitory activity of the fractions on complement-dependent cytotoxicity of D23 TBS, diluted 1/100, for D23 cells. 25 mg protein in 1 ml was applied to the column. The inhibitory activity was calculated from cytotoxicity data corrected by subtracting percentage $^{51}$Cr release in medium controls (12·1%). Percentage $^{51}$Cr release with D23 TBS diluted 1/100 was 45·7% ± 1.5%. The column was calibrated with Blue Dextran (Vo), ferritin, human IgG, bovine serum albumin, cytochrome C and tryptophane (Ve). (——), absorbancy at 280 nm; (× --- ×), percentage inhibition of cytotoxicity.

longed, a D23 KCl extract was fractionated on a Sepharose CL-4B column in the presence of 3M KCl (Fig. 1). The fractions were dialysed separately against distilled water in the cold, and were then frozen and lyophilized. The lyophilized material was suspended in PBS and then tested for inhibition on a volume basis (Fig. 1). The main inhibition was found in Fractions 22–29. There was also a marked inhibition in the HMW Fractions 17 to 19. This latter activity however, varied from fractionation to fractionation, and probably consisted of aggregates of the inhibitory components found in the LMW regions. The highest specific inhibition (inhibition per absorbancy at 280 nm) was found in Fractions 22 and 23.

The polypeptide pattern of the different fractions was monitored by means of SDS–PAGE. Although Fractions 13–16 gave a high absorbancy at 280 nm, very little protein was recovered from these, as established both by SDS–PAGE and by Lowry's protein determination. However, this HMW material had an absorbancy maximum at 260 nm and probably consisted of nucleic acids. This was confirmed in experiments where hepatoma D23 ENP fractions were digested with DNase, subsequently extracted with 3M KCl and applied to column chromatography on Sepharose CL-4B. In contrast to a KCl-extract of an untreated D23 ENP, the extract of the DNase-treated ENP showed no absorbancy-peak in the HMW region.

Fractionation of KCl extracts of liver and kidney on Sepharose CL-4B revealed the inhibitory activity chromatographing in the same mol. wt regions as those of D23 KCl extracts.

To elucidate further the nature of the antigen(s) responsible for this tumour-associated immune response, 3M-KCl extracts of D23 ENP were precipitated with 10% TCA. The TCA-soluble and TCA-insoluble fractions were tested for inhibition of D23 TBS cytotoxicity against D23 cells (Table II). Most of the inhibitory activity was precipitated with TCA, though some activity was also found in the TCA-soluble fraction. This incidates that the antigenicity in the 3M-KCl extracts is mainly associated with proteins. On the other hand, heating D23 KCl extracts for 15 min at 100°C did not affect the inhibitory activity of these extracts (data not shown) suggesting the nonprotein nature of the antigens.

TABLE II.—*Inhibition of D23 TBS cytotoxicity against D23 cells with TCA-treated D23 KCl extracts*

| | % Inhibition* with | | |
|---|---|---|---|
| Extract dilution | D23 KCl† | D23 KCl TCAsol‡ | D23 KCl TCAinsol§ |
| 1/5 | 84·9 | 25·9 | 68·6 |
| 1/50 | 46·3 | 3·0 | 43·3 |
| 1/500 | 43·9 | 0 | 35·5 |

* Percentage inhibition was calculated according to Materials and Methods. Percentage $^{51}$Cr release in medium control was 18·0 ± 1·0%, with WRS (diluted 1/100) 18·8 ± 2·4% and with D23 TBS (diluted 1/100) 34·6 ± 0·5%.

† 3M-KCl extract of the D23 hepatoma (5 mg protein/ml) tested at the dilutions indicated.

‡ The TCA-soluble portion, after TCA precipitation of 5 mg D23 KCl extract protein, in 1 ml PBS, was tested at the dilutions indicated.

§ The TCA-precipitated portion of the same procedure, in 1 ml PBS, was tested at the dilutions indicated.

Digestion of D23 ENP with different enzymes was performed to obtain further information of the nature of the molecules containing or being associated with the antigenicity. D23 ENP was treated with a panel of enzymes and then tested for inhibition of D23 TBS cytotoxicity against D23 cells (Fig. 2). β-Glucosidase, DNase, papain and trypsin were all without effect on the inhibitory activity of D23 ENP, whilst the phospholipases $A_2$, C and D consistently abrogated it. In the experiments presented, neuraminidase treatment increased the inhibitory activity of D23 ENP. The significance of this is not known. Moreover, neuraminidase treatment had no effect in 3 similar experiments. The relative effect of treatments with the different phospholipases varied between experiments, but a decrease in the inhibitory activity of these membranes was seen in all 4 experiments.

The influence of the enzyme treatments on the polypeptide pattern of D23 ENP was investigated in SDS–PAGE (Fig. 3). Neuraminidase, $\beta$-glucosidase and DNase did not alter the polypeptide pattern from untreated D23 ENP. Phospholipase C digestion of D23 ENP removed some polypeptides, whilst papain and trypsin digestion destroyed most of the native polypeptide pattern and several LMW polypeptides appeared (Phospholipase $A_2$ or D-digested D23 ENP were not tested in this gel).

The lack of correlation between protein content and antigenic activity in the experiments with D23 KCl extracts, as well as the heat stability of the antigenicity and its lack of sensitivity to proteolytic digestion, prompted us to investigate the possible lipid nature of the antigen(s). D23 ENP was subjected to different extractions with organic solvents. Extractions with n-butanol or chloroform/methanol gave very similar results. Thus, the antigenicity was recovered in the organic solvent and interphases, but no

FIG. 2.—Inhibition with enzyme-digested D23 ENP of D23 TBS cytotoxicity against D23 cells. (a) Inhibition with D23 ENP treated with $\beta$-glucosidase (0·05% in PBS) ×---×, neuraminidase (0·02% in PBS) Y---Y, DNase (0·25% in PBS) +---+, trypsin (0·25% in 1mM $NaHCO_3$, 1mM $CaCl_2$, pH = 7·6) *---*, papain (0·02% in 0·05M Tris-buffer, 0·1M L-cysteine, pH = 7·0) ·----·, untreated D23 ENP ×—×. (b) Inhibition with D23 ENP treated with: phospholipase $A_2$ (0·02% in 0·05M Tris-buffer, 2mM $CaCl_2$, pH = 8·5) ×---×, phospholipase C (0·10% in 0·05M Tris-buffer, 2mM $CaCl_2$, pH = 7·3) Y---Y, phospholipase D (0·10% in 0·2M acetate-buffer, 50mM $CaCl_2$, pH = 5·6) +---+, untreated D23 ENP ×—×. The membranes were tested at various protein concentrations. Percentage $^{51}Cr$ release in medium control was 12·9% ± 0·6%, with WRS diluted 1/100, 12·7% ± 0·3% and with D23 TBS diluted 1/100, 52·2% ± 0·1%.

inhibition was found in the water phase (Fig. 4). No anticomplementarity was detected in the fractions. The 3M-KCl extracted antigenicity was also found to be soluble in the solvent when extracted with chloroform/methanol. No difference in this respect was found between the PBS-soluble and the PBS-insoluble anti-

FIG. 3.—SDS-PAGE of D23 ENP after digestion with neuraminidase (1), β-glucosidase (2), DNase (3), phospholipase C (4), trypsin (5) and papain (6). The dot in Slot 4 indicates a polypeptide corresponding to phospholipase C. The numbers on the left indicate molecular weight × $10^{-3}$.

genicity of the D23 KCl extracts. These results suggest the lipid nature of the target antigen(s) for the cytotoxic IgM antibodies in D23 TBS.

### DISCUSSION

3M-KCl extracts of tumours and some normal tissues were found to contain components that abrogate the complement-dependent cytotoxicity of D23 TBS against D23 cells. Since it has been reported that normal tissues contain anticomplementary activity (Ormod & Miller, 1978) it was important to establish that the inhibition of cytotoxicity by our KCl or organic-solvent extracts was not due to such activity. No anticomplementarity was found with the extracts tested, indicating that in the concentration ranges used, the abrogation of cytotoxicity was caused by inhibition of antigen/antibody reactions.

KCl treatment of hepatomas yields extracts that contain a heterogeneous population of molecules, many of which apparently retain their antigenicities. Such extracts of many tumours (Meltzer *et al.*, 1971; Leonard *et al.*, 1975; Barra *et al.*, 1977) and particularly of the chemically induced rat hepatomas, have been shown to contain antigens that inhibit the cell-mediated cytotoxicity to the tumour (Zöller *et al.*, 1976; 1977), antigens that react with circulating antibodies in serum from tumour-bearing animals (Zöller *et al.*, 1976) and antigens that can induce immunoprotection against the tumour (Price *et al.*, 1978). Whether these effects are caused by the same antigens is not known. However, the antigens described by these authors have been reported to comprise tumour-specific as well as embryonic tumour-associated components (Zöller *et al.*, 1976, 1977; Price *et al.*, 1978). Although we obtained good inhibition of cytotoxicity with both D23 hepatoma extracts and with hepatoma cell-sap fractions (Lando *et al.*, 1979) known to contain embryonic antigens (Baldwin *et al.*, 1974) the involvement of such antigens in our systems seems of minor significance, since cytotoxicity was efficiently abrogated by absorption with KCl extracts of normal adult rat tissues. Absorption of TBS with foetal liver cells also failed to affect cytotoxicity (Lando *et al.*, 1977).

In a complement-dependent cytotoxicity assay very similar to ours, Price & Baldwin (1977) also studied the reaction of D23 TBS with D23 hepatoma cells. As in our case (Lando *et al.*, 1977, 1980*b*) the cytotoxicity described by these authors was due to HMW antibodies, most probably IgM (Price & Baldwin, 1977). Since

FIG. 4.—Inhibition with organic-solvent-extracted D23 ENP of complement-dependent cytotoxicity of D23 TBS against D23 cells. First striped bar, percentage $^{51}$Cr release with WRS diluted 1/100; second striped bar, percentage $^{51}$Cr release with D23 TBS diluted 1/100. 25 mg D23 ENP (determined by the method of Lowry) in PBS was extracted with n-butanol (see Materials and Methods). Percentage $^{51}$Cr release in medium control was 9·2% ± 0·2%. 1st group of open bars, percentage $^{51}$Cr release of D23 TBS diluted 1/100 mixed with the butanol-soluble fraction of D23 ENP diluted 1/5, 1/25, 1/50,1/100 and 1/200. 2nd and 3rd groups of open bars, the corresponding results using the interphase and water phase corresponding to the same butanol extracts.

these D23 TBS did not lyse two other hepatomas (D192 and D202) and since absorption with a third hepatoma (D30) did not abrogate cytotoxicity, it was concluded that these antibodies were directed against a tumour antigen specific for D23. However, our cytotoxicity experiments with D23 and other hepatoma TBS (including D30 TBS and D202 TBS) in reciprocal combinations suggest that the major cytotoxicity in this system is directed to one or several antigens shared by the tumours, but expressed to different degrees (Lando *et al.*, 1980*a*). This was confirmed when 3M-KCl extracts from 5 different hepatomas and several normal adult rat tissues were tested for inhibition (data not shown).

The cytotoxicity-inhibiting activity in 3M-KCl extracts is distributed over a broad range of high mol. wt. Similar results have been reported by Leonard *et al.* (1975), who investigated the tumour-specific antigenicity in 3M-KCl extracts from line-10 guinea-pig hepatoma. The heterogeneous distribution of the activity found after Sephadex G-200 fractionation in the presence of 2M KCl was thought to be due to either proteolysis of the antigen or to chemical heterogeneity. Since the results presented herein show that the antigen(s) present in the extracts are probably lipids, the heterogeneity found could be explained by micelle formation or the association of the lipid molecules with proteins. Whether or not the antigenicity in the 3M-KCl extracts is caused by the lipid molecules only, remains to be established. The presence of the antigenicity in the cell-sap fraction of D23 hepatoma (Lando *et al.*, 1979) and in the PBS-soluble fraction of 3M-KCl extracts indicated that the antigenicity appears both in water-soluble and insoluble forms.

Both the water-soluble and insoluble antigen fractions are, however, able to completely abrogate the antibody activity, indicating that these fractions contain the same or similar antigen(s). Further, when a complete KCl extract, a PBS-soluble fraction of a KCl extract or a cell-sap fraction is submitted to extraction with organic solvents the antigenicity is recovered only in the organic solvent and not in the water. Thus, the water solubility of the antigenicity can be explained either by association of the antigenic molecules with hydrophobic regions of the water-soluble proteins, or by the formation of micelles by the antigen molecules.

The inhibitory antigens are probably largely of membrane origin, as the cytotoxic reactions take place at the cell surface, and as the antigens are found in isolated liver plasma membranes (Lando *et al.*, 1977). According to our enzyme-digestion experiments the inhibitory antigens seem to be resistant to proteolysis, but susceptible to phospholipase. The mechanism of the abrogation of the D23 ENP inhibition by the phospholipases is not yet known. The enzyme treatment might solubilize the antigen(s) as has been reported for membrane proteins by Raftell & Blomberg (1974). However, as will be shown elsewhere (Lando *et al.*, submitted) the antigen(s) are recovered in the phospholipid-containing fractions after column chromatography on silica gel, and after preparative thin-layer chromatography, suggesting their phospholipid nature. Thus, it is likely that the phospholipases directly digest the antigen(s) and thereby abrogate the inhibitory activity of D23 ENP.

We thank Margaretha Hagstedt and Ingegärd Andersson for excellent technical assistance. This work was supported by Grant No. 642-B80-07X from the Swedish Cancer Society.

### REFERENCES

BALDWIN, R. W. (1964) Modification of cell antigens during aminoazo-dye carcinogenesis in a rat liver. *Br. J. Cancer*, **18**, 285.

BALDWIN, R. W. & BARKER, C. R. (1967) Demonstration of tumour-specific humoral antibody against aminoazo-dye-induced rat hepatoma. *Br. J. Cancer*, **21**, 793.

BALDWIN, R. W., BOWEN, J. G. & PRICE, M. R. (1973*a*) Detection of circulating hepatoma D23 antigen and immune complexes in tumour bearer serum. *Br. J. Cancer*, **28**, 16.

BALDWIN, R. W., GLAVES, D. & VOSE, B. M. (1974) Differentiation between the embryonic and tumour specific antigens on chemically induced rat tumours. *Br. J. Cancer*, **29**, 1.

BALDWIN, R. W., HARRIS, J. R. & PRICE, M. R. (1973*b*) Fractionation of plasma membrane-associated tumour-specific antigen from an aminoazo-dye-induced rat hepatoma. *Int. J. Cancer*, **11**, 385.

BARRA, Y., ASTIER, A.-M. & MEYER, G. (1977) Isolation of polyoma virus-induced surface antigens in hamster cells: Potassium chloride solubilization and differential precipitation. *J. Natl Cancer Inst.*, **58**, 721.

CHUA, N.-H. & BENNOUN, P. (1975) Thylakoid membrane polypeptides of *Chlamydomonas rienhardtii*: Wildtype and mutant strains deficient in photosystem II reaction center. *Proc. Natl Acad. Sci. U.S.A.*, **72**, 2175.

COHEN, R. S., BLOMBERG, F., BERZINS, K. & SIEKEVITZ, P. (1977) The structure of postsynapatic densities isolated from dog cerebral cortex. I. Overall morphology and protein composition. *J. Cell Biol.*, **74**, 181.

HÖFFKEN, K., PRICE, M. R., MCLAUGHLIN, P. F., MOORE, V. E. & BALDWIN, R. W. (1978*a*) Circulating immune complexes in rats bearing chemically induced tumors. I. Sequential determination during the growth of tumors at various body sites. *Int. J. Cancer*, **21**, 496.

HÖFFKEN, K., PRICE, M. R., MOORE, V. E. & BALDWIN, R. W. (1978*b*) Circulating immune complexes in rats bearing chemically induced tumors. II. Characterization of sera from different stages of tumor growth. *Int. J. Cancer*, **22**, 576.

LANDO, P., BLOMBERG, F., BERZINS, K. & PERLMANN, P. (1979) Tumor-induced cytotoxic antibodies in the serum of hepatoma-bearing rats. In *Protides of the Biological Fluids*, Vol. 27. Ed. Peeters. Oxford: Pergamon Press. p. 165.

LANDO, P., BLOMBERG, F., BERZINS, K. & PERLMANN, P. (1980*a*) Tumor-associated cytotoxicity of autoantibodies in sera from rats with chemically induced hepatomas. *Cancer Res.*, **40**, 1671.

LANDO, P., BLOMBERG, F., RAFTELL, M., BERZINS, K. & PERLMANN, P. (1977) Complement-dependent cytotoxicity against hepatoma cells mediated by IgM antibodies in serum from tumor-bearing rats. *Scand. J. Immunol.*, **6**, 1081.

LANDO, P., GABRIEL, J., BERZINS, K. & PERLMANN, P. (1980*b*) Determination of the immunoglobulin class of complement-dependent cytotoxic antibodies in serum of D23 hepatoma-bearing rats. *Scand. J. Immunol.*, **11**, 253.

LEONARD, E. J., RICHARDSON, A. K., HARDY, A. S. & RAPP, H. J. (1975) Extraction of tumor-specific antigen from cells and plasma membranes of Line-10 hepatoma. *J. Natl Cancer Inst.*, **55**, 73.

MELTZER, M. S., LEONARD, E. J., RAPP, H. J. & BORSOS, T. (1971) Tumor-specific antigen solubilized by hypertonic potassium chloride. *J. Natl Cancer Inst.*, **47**, 703.

NEVILLE, D. M. JR (1971) Molecular weight determinations of protein-dodecyl sulfate complexes by gel electrophoresis in a discontinuous buffer system. *J. Biol. Chem.*, **246**, 6328.

ORMOD, D. J. & MILLER, T. E. (1978) Complement-mediated immune mechanisms in renal infection. I. Effect of renal tissue *in vitro*. *Clin. Exp. Immunol.*, **33**, 107.

PRICE, M. R. & BALDWIN, R. W. (1977) Tumor-specific complement-dependent serum cytotoxicity against a chemically induced rat hepatoma. *Int. J. Cancer*, **20**, 284.

PRICE, M. R., PRESTON, V. E., ROBINS, R. A., ZÖLLER, M. & BALDWIN, R. W. (1978) Induction of immunity to chemically-induced rat tumours by cellular or soluble antigens. *Cancer Immunol. Immunother.*, **3**, 274.

RAFTELL, M. & BLOMBERG, F. (1974) Enzyme polymorphism in rat-liver microsomes and plasma membranes. 2. An immunochemical comparison of enzyme-active antigens solubilized by detergents, papain or phospholipases. *Eur. J. Biochem.*, **49**, 31.

ROBINS, R. A. & BALDWIN, R. W. (1974) Tumour-specific antibody neutralization of factors in rat hepatoma-bearer serum which abrogate lymph-node-cell cytotoxicity. *Int. J. Cancer*, **14**, 589.

STARK, J. M., MATTHEWS, N. & LOCKE, J. (1980) Immunogenicity of lipid-conjugated antigens. II. Anti-complementary activity and antigen trapping in the spleen. *Immunol.*, **39**, 353.

ZÖLLER, M., PRICE, M. R. & BALDWIN, R. W. (1976) Inhibition of cell-mediated cytotoxicity to chemically induced rat tumors by soluble tumor and embryo cell extracts. *Int. J. Cancer*, **17**, 129.

ZÖLLER, M., PRICE, M. R. & BALDWIN, R. W. (1977) Evaluation of $^{51}Cr$ release for detecting cell-mediated cytotoxic response to solid chemically induced rat tumours. *Br. J. Cancer*, **35**, 834.